# A single dose of SAAVI MVA-C reboosts rhesus macaques after more than 3 years post DNA-MVA prime-boost vaccination

**DOI:** 10.1186/1742-4690-9-S2-P32

**Published:** 2012-09-13

**Authors:** GK Chege, W Burgers, T Muller, EG Shephard, C Williamson, A Williamson

**Affiliations:** 1 University of Cape Town, Cape Town, South Africa

## Background

We have previously reported induction of robust immune responses in rhesus macaques following a prime boost immunization with candidate HIV-1 vaccines, SAAVI DNA-C (DNA) and SAAVI MVA-C (MVA). These vaccines are already in clinical evaluation. In the current study, we investigated whether re-boosting these animals with a single MVA inoculation after more than 3 years was sufficient to restore previous magnitudes of HIV-specific immune responses.

## Methods

Seven rhesus macaques which had been vaccinated with three doses of DNA vaccine (4mg DNA/dose) and two doses of MVA (10^9^ pfu MVA/dose) in a past study, >3 years previously, were re-boosted with a single dose of MVA. HIV-1-specific responses were quantified in the peripheral blood using an IFN-gamma ELISPOT assay.

## Results

A peak magnitude of response (1146±240 sfu/10^6^ PBMC) was reached 1 week after vaccination with the first dose of MVA. The second MVA inoculation did not increase these responses which declined to undetectable levels by 1 year post vaccination. After re-boosting with MVA after 3.5 years post the second MVA, all animals responded, with a peak response (1824±672 sfu/10^6^ PBMC) being reached 1 week after vaccination. Although the mean magnitude of the second peak was not significantly higher than the one seen in the first peak, boosting of responses in 3 of 7 animals with an apparent broadening of the breadth of responses was observed.

## Conclusion

These preliminary data suggest a long-term preservation of vaccine memory following a prime-boost vaccination regimen with SAAVI DNA-C and SAAVI MVA-C vaccines.

